# Healthy Photosynthetic Mechanism Suggests ISR Elicited by *Bacillus* spp. in *Capsicum chinense* Plants Infected with PepGMV

**DOI:** 10.3390/pathogens10040455

**Published:** 2021-04-10

**Authors:** Blancka Yesenia Samaniego-Gámez, René Garruña, José M. Tun-Suárez, Oscar A. Moreno-Valenzuela, Arturo Reyes-Ramírez, Raúl Enrique Valle-Gough, Carlos Enrique Ail-Catzim, Lydia Toscano-Palomar

**Affiliations:** 1 Institute of Agricultural Sciences, Autonomous University of Baja California, Carretera Blvd, Delta s/n Ejido Nuevo León, Valle de Mexicali, Baja California C.P. 21705, Mexico; raul.valle@uabc.edu.mx (R.E.V.-G.); carlos.ail@uabc.edu.mx (C.E.A.-C.); 2 National Technological Institute of Mexico—Technological Institute of Conkal, Av. Tecnológico s/n, Conkal, Yucatán C.P. 97345, Mexico; jose.tun@itconkal.edu.mx (J.M.T.-S.); arturo.reyes@itconkal.edu.mx (A.R.-R.); 3 CONACYT—Technological Institute of Conkal, Av. Tecnológico s/n, Conkal, Yucatán C.P. 97345, Mexico; 4 Scientific Research Center of Yucatan A.C., Department of Plant Biochemistry and Molecular Biology, Calle 43 No. 130, Colonia Chuburná de Hidalgo, Mérida, Yucatán C.P. 97200, Mexico; oamv@cicy.mx; 5 National Technological Institute of Mexico—Technological Institute of Mexicali, Av. Instituto Tecnológico s/n, Plutarco Elías Calles, Mexicali, Baja California C.P. 21376, Mexico; toscano.lydia@itmexicali.edu.mx

**Keywords:** chlorophyll fluorescence, gas exchange, geminiviridae, PGPR

## Abstract

The aim of this study was to evaluate the effect of inoculation with *Bacillus* spp. isolates on the photosynthetic apparatus of *Capsicum chinense* plants infected with PepGMV. In vitro and greenhouse experiments were performed to evaluate whether the inoculation improved plants’ performance through the increase in photosynthetic efficiency to control PepGMV. The results showed that despite PepGMV infection, the plants inoculated with some isolates of *Bacillus* spp. had a healthy photosynthetic mechanism, as the photochemical parameters and gas exchange increased. The maximum photochemical quantum yield of PSII (Fv/Fm) of plants with PepGMV and inoculated with *Bacillus* isolates (M9, K46, and K47) increased (7.85, 7.09, and 7.77%, respectively) with respect to uninoculated controls. In inoculated plants, the CO_2_ assimilation rate increased and the transpiration rate decreased, therefore indicating an increased water use efficiency. This effect was reflected by the less severe symptoms caused by PepGMV in the plants obtained from seeds inoculated with different *Bacillus* spp. Plants inoculated with K47 isolates showed an increase in fruit yield and quality. This study suggests that it is possible to protect, at the greenhouse level, *C. chinense* plants from PepGMV through selected rhizobacteria inoculation.

## 1. Introduction

Diseases caused by plant pathogenic viruses are common in agricultural crops and cause high economic losses [1]. The genus *Begomovirus* is the most widespread and diverse worldwide. *Pepper golden mosaic virus* (PepGMV) is a Begomovirus that is widely distributed in Mexico, where it is considered a major viral pathogen in pepper and other economically important solanaceous crops, such as tomato and tobacco [2]. PepGMV, a bipartite virus, has been reported to be restricted to the vascular tissue, causing bright yellow mosaic symptoms on leaves. These symptoms are associated with the twisting and distortion of leaves and fruits, stunted plants, and reduced yield. PepGMV is transmitted by whiteflies (*Bemisia tabaci* Gennadius B biotype) [3,4]. Physiological studies have shown that many viral diseases decrease photosynthesis due to their reduction in photosynthetic pigments [5,6,7]. In addition, *Cucumber mosaic virus* (CMV) infection caused an alteration in electron transport during photosynthesis and respiration in cucumber and tomato, affecting antioxidant systems and leading to oxidative stress in some organelles [7]. Similarly, in apricot cultivars infected with *Plum pox virus* (PPV), the Photosystem II (PSII) was directly or indirectly affected [8,9]. In *Nicotiana benthamiana* plants infected with tobamoviruses, it was observed that infection reduced the PSII electron transport, affecting the polypeptide composition of the oxygen-evolving complex [10].

Recently there has been an increase in the use of products based on beneficial microorganisms to manage many plant diseases [11,12]. Studies have indicated the importance of different rhizobacteria, such as *Azospirillum, Azotobacter, Gluconacetobacter, Pseudomonas,* and *Bacillus*, as alternative tools for biological control and growth promotion [13,14]. Many isolates of *Bacillus* have been reported as growth promoters, regulating a number of plant physiology parameters. Various properties have been attributed to these bacteria, such as IAA production; N_2_ fixation; and siderophore production, including schizokinen, phosphate solubilizing, and the secretion of acid phosphatases and phytases [14,15,16,17,18]. Some of these bacteria may also produce different compounds with antagonistic and pathogenic properties, such as extracellular antibiotics—i.e., bacilomycin, iturin, mycosubtilin, and zwittermicin—or lytic enzymes, such as chitinase, protease, and β-1,3 glucanase. Other properties include the competition for nutrients in the host and Induced Systemic Resistance (ISR), achieved by eliciting the jasmonic acid and ethylene signaling pathways [12,19]. In infections caused by plant viruses, it has been shown that inoculation with *Bacillus* spp. promotes ISR, favoring a decrease in viral symptoms. This effect is attributed to defense gene expression, decreasing viral replication and thus sustaining plant growth [20].

Most studies on ISR elicited by *Bacillus* spp. have been directed at the secondary metabolites produced. However, it has been observed that *Bacillus* spp. could catalyze physiological changes in plants that have not yet been fully investigated [21,22]. Some authors [23,24] studied the effect of *Bacillus* spp. on the photosynthetic system of *Capsicum chinense* (Habanero pepper), and others showed that *Bacillus* spp. inoculation improved the photosystem II efficiency and enhanced photosynthesis in pepper plants [25]. In this sense, we hypothesized that inoculation with *Bacillus* spp. in pepper plants infected with viruses would sustain photosynthesis and thus yield. Therefore, the aim of this study was to evaluate the effect of inoculation with *Bacillus* spp. on the photosynthetic apparatus of *C. chinense* plants infected with PepGMV.

## 2. Results

### 2.1. Infectivity, Viral Detection and Symptoms Severity

Both in the growing room and in the greenhouse, the plants obtained from seeds inoculated with *Bacillus* isolates showed a lower severity of symptoms caused by PepGMV during the entire course of the experiment, indicating some tolerance to the virus (Table 1). PepGMV was detected by PCR amplification in all plants infected with biolistics, whereas it was not detected in control plants (bombarded only with gold particles, without the virus) (Table 1). At 9 dpi, an attenuation of symptoms was observed in plants inoculated with *Bacillus* isolates. Plants that were not inoculated were statistically similar to those inoculated with K46 in severity, but different from those inoculated with M9 and K47 and the untreated control. However, at 15 dpi the plants inoculated with M9, K46, and K47 isolates had a statistically higher severity than the control, but a lower one than that of PepGMV-inoculated plants (Table 1).

**Figure 1:**
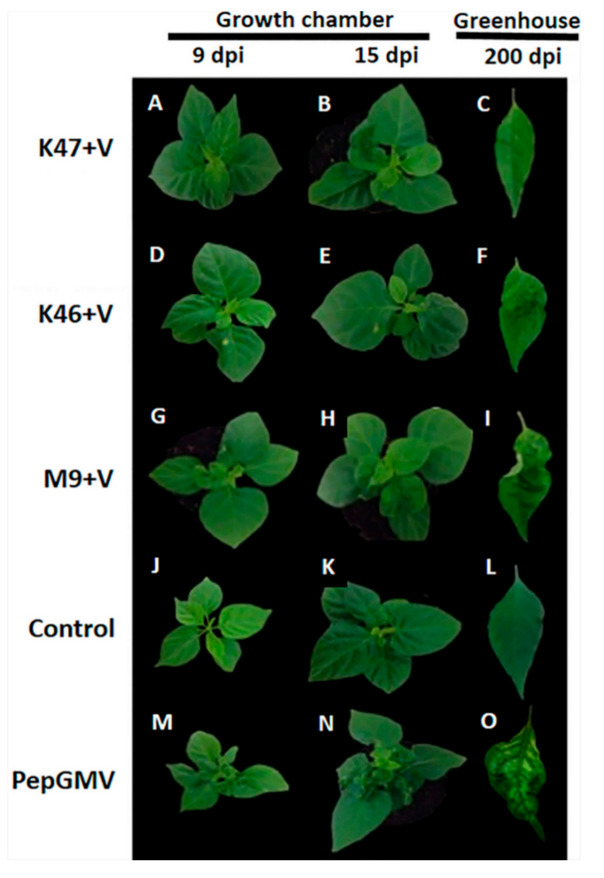
Effect of pepper inoculation with *Bacillus subtilis* isolates K47, *Bacillus cereus* spp. isolates K46, *Bacillus* spp. isolates M9, and infection with PepGMV at 9 (**A**,**D**,**G**), 15 (**B**,**E**,**H**), and 200 (**C**,**F**,**I**) days post-infection (dpi). Effect of PepGMV infection alone at 9 (**M**), 15 (**N**), and 200 (**O**) dpi. Control plants (bacteria not inoculated and no PepGMV infection) at 9 (**J**), 15 (**K**), and 200 (**L**) dpi. *n* = 30.

Both at 9 and 15 dpi, plants infected with PepGMV had some moderate yellow mosaic, wrinkle symptoms, deformation of leaves, and dwarfing (Figure 1). Subsequently, the plants were transferred to a greenhouse under controlled conditions. At 200 dpi in the greenhouse, symptoms of light golden mosaics and some deformation of leaves in plants with *Bacillus* spp. isolates K47, K46, and M9 were observed (Figure 1C,F,I). The plants infected with PepGMV without *Bacillus* spp. were more affected than those inoculated with the *Bacillus* isolates (Figure 1O). The control plants, without viruses or rhizobacteria, did not show symptoms (Figure 1L). No symptom was observed during the experimental course (Figure 1J,K,L).

### 2.2. Physiological Responses

**Figure 2:**
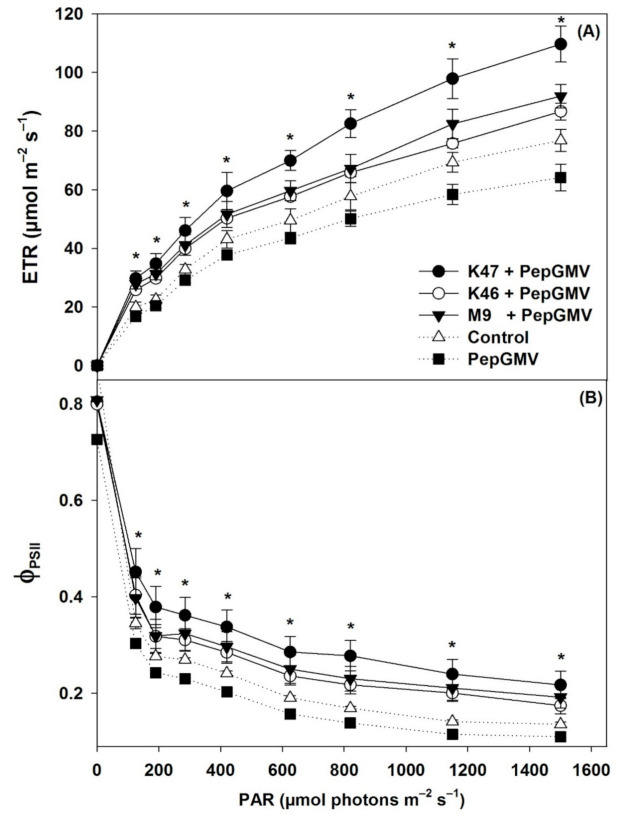
Response curves of electron transport rate (**A**) and effective photochemical quantum yield (**B**) of photosystem II to photosynthetic photon flux density in *Capsicum chinense* plants infected with PepGMV and inoculated with *Bacillus subtilis* K47, *B. cereus* K46, *Bacillus* spp. M9; and plants infected with PepGMV not inoculated. Control = plants not inoculated and not infected. Data are means ± SE. * = statistically significant (ANOVA, *p* ≤ 0.05, *n* = 30).

The electron transport rate of photosystem II (ETR_PSII_) decreased in plants with PepGMV (non-inoculated with the *Bacillus* spp.) with respect to the control plants, but ETR_PSII_ increased in all treatments with *Bacillus* spp. isolates to increase the photosynthetic photon flux density. In plants inoculated with K47, the ETR_PSII_ increased 30 and 41.6% with respect to the control and plants infected with PepGMV, respectively (Figure 2A). The effective photochemical quantum yield of PSII (Φ_PSII_) was statistically higher in plants inoculated with the *Bacillus* isolates (K47, M9, and K46). Plants infected with PepGMV without the inoculation of bacteria had lower values of photosynthetic photon flux density (Figure 2B).

**Figure 3:**
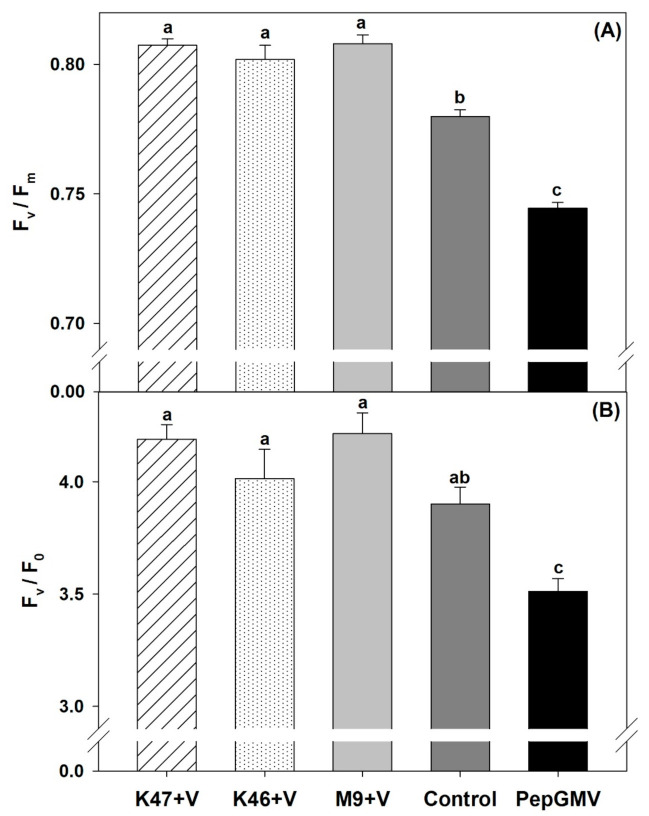
Maximum photochemical quantum yield (**A**) and potential activity (**B**) of PSII in *Capsicum chinense* plants infected with PepGMV and inoculated with *Bacillus subtilis* K47, *B. cereus* K46, *Bacillus* spp. M9; and plants infected with PepGMV not inoculated. Control are plants not inoculated and not infected. All plants inoculated with bacteria were infected with PepGMV. Data are means ± SE. Different letters represent statistically significant differences (Tukey, α = 0.05, *n* = 30).

The ratio of variable to maximum fluorescence or maximum photochemical quantum yield of PSII (F_v_/F_m_) was statistically higher (ANOVA, *p* ≤ 0.05) in plants inoculated with M9, K46, and K47 in 7.85, 7.09, and 7.77%, respectively, than in PepGMV–infected plants (Figure 3A). The potential activity of PSII (F_v_/F_0_) showed a statistically similar trend to F_v_/F_m_. Values for plants inoculated with M9 and K47 isolates were 16.76 and 16.05% higher than for PepGMV–infected plants, but the control plants were statistically similar to those inoculated with the *Bacillus* isolates (Figure 3B).

**Figure 4:**
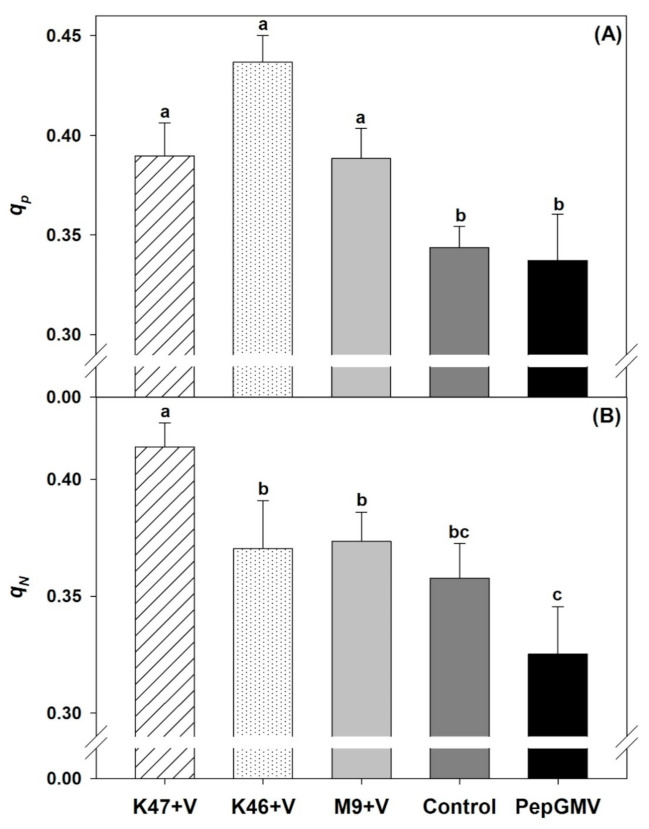
Photochemical quenching (**A**) and non-photochemical quenching (**B**) in *Capsicum chinense* plants infected with PepGMV and inoculated with *Bacillus subtilis* K47, *B. cereus* K46, *Bacillus* spp. M9; and plants infected with PepGMV not inoculated. Control = plants not inoculated with *Bacillus* spp. and not infected by PepGMV. Data are means ± SE. Different letters represent statistically significant differences (Tukey, α = 0.05, *n* = 30).

Plants inoculated with M9, K47, and K46 isolates had a statistically higher coefficient of photochemical quenching (qP) than the control and PepGMV–infected plants (Figure 4A). Additionally, non-photochemical quenching (NPQ) showed a statistically similar trend to the coefficient of photochemical quenching (qP), but the control plants were statistically similar to those inoculated with the *Bacillus* isolates (Figure 4B).

**Figure 5:**
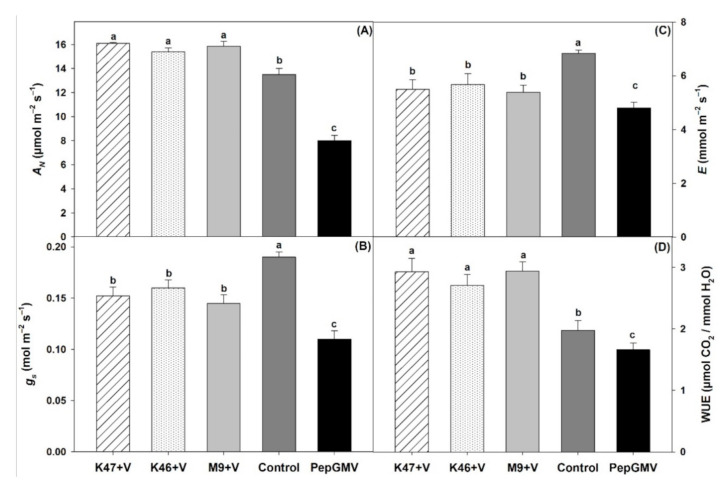
CO_2_ Assimilation rate (**A**), stomatal conductance (**B**), transpiration (**C**), and water use efficiency (**D**) in *Capsicum chinense* plants infected with PepGMV and inoculated with *Bacillus subtilis* K47, *B. cereus* K46, *Bacillus* spp. M9; and plants infected with PepGMV not inoculated. Control = plants not inoculated with bacteria and not infected by PepGMV. Data are means ± SE. Different letters represent statistically significant differences (Tukey, α = 0.05, *n* = 30).

Inoculation with *Bacillus* spp. increased the CO_2_ assimilation in the treated plants compared to those that were uninoculated (Figure 5A). The CO_2_ assimilation rate in the plants inoculated with *Bacillus* and the control was statistically higher than in the PepGMV–infected plants (Figure 5A). Plants inoculated with *Bacillus* K47, K46, and M9 showed values increased by 22.10, 26.31, and 18.42% in comparison to virus-infected plants. However, nevertheless, plants inoculated with *Bacillus* spp. showed both decreased stomatal conductance (Figure 5B) and transpiration (Figure 5C) compared to the control plants. Additionally, the stomatal conductance and transpiration in plants inoculated with *Bacillus* spp. and the control plants were statistically higher than in the PepGMV–infected plants (Figure 5B,C). On the other hand, *Bacillus* K47, K46, and M9 enhanced the water use efficiency by 43.12, 35.70, and 43.52%, respectively, compared to the PepGMV–infected plants (Figure 5D).

### 2.3. Yield of Plants with Bacillus spp.-C. chinense -PepGMV in Greenhouse

*Bacillus* inoculation in seeds favored the greenhouse production of *C. chinense* infected with PepGMV (Table 2). Symptoms of PepGMV were observed 200 days after infection, with a lower severity shown in plants inoculated with the three *Bacillus* spp. The plants without the *Bacillus* inoculation showed more severe symptoms caused by PepGMV throughout the production cycle. Those with virus and *Bacillus* K46 or K47 showed the highest yields (Tukey, α = 0.05).

The yields of plants with rhizobacteria and the virus were statistically higher than those of plants that were not inoculated with *Bacillus* and the viral disease. Similarly, it was found that the fruits of plants with the *Bacillus* sp. K47-PepGMV treatment showed the highest weight. It was also observed that the plants inoculated with *Bacillus* spp. M9 and PepGMV or *Bacillus* sp. K46 and PepGMV produced fewer fruits per plant compared to both controls. However, the fruits produced were heavier than those of the control plants (Table 2). Plants inoculated with *Bacillus* spp. produced fruits whose weight was higher than that of uninoculated or PepGMV-inoculated plants (Table 2). This suggests that inoculation with *Bacillus* spp. sustained the plant performance and increased the fruit quality, despite the presence of PepGMV.

## 3. Discussion

In this study, it was observed that biolistic inoculation caused symptoms such as golden mosaics, deformed leaves, and dwarfing in the plants grown in culture chambers. These symptoms have been observed in other studies using the same technique and PepGMV as a model [3,26]. However, in plants previously inoculated with different *Bacillus* isolates and infected with PepGMV, the disease severity was statistically lower when compared with plants without inoculation with rhizobacteria. At the symptom level, it was observed that plants inoculated with *Bacillus* spp. and infected with the virus showed a lower viral accumulation, incidence, and severity. Additionally, inoculated plants had a better photosynthetic performance. The mentioned could be attributed to a *Bacillus*-mediated ISR or viral replication inhibition or dilution due to increased plant growth [27,28].

Investigations have evaluated the inoculation effect of *Bacillus* sp. in greenhouses and the open field, showing a reduction in the severity and incidence of other viral diseases at 28 and 40 dai with *Tobacco mosaic virus* (TMV), *Tomato mottle virus* (ToMoV), and *Cucumber mosaic virus* (CMV). The ISR induction was attributed to inoculation with *Bacillus* spp. [27,29]. In a study on CMV, it was observed that, during the second year of production, the disease incidence and severity levels were higher than in the first year. The reduced effectiveness of the rhizobacteria treatments was possibly caused by the increased levels of CMV naturally transmitted by aphids. Moreover, the plants may have been infected naturally with CMV isolates against which the *Bacillus* spp. used were not effective [30].

In our study, we observed at 200 dpi signs of wrinkling and light golden mosaics in plants previously inoculated with *Bacillus* K47, K46, and M9. However, plants inoculated with K47 showed a statistically low severity of PepGMV symptoms, with higher yields and fruit weight compared to inoculation with other *Bacillus* spp. isolates. This variability in the effect of inoculation with *Bacillus* spp. could depend on the bacteria isolates, the host plant, and the interactions with the pathogen used [11,21]. Furthermore, the induced resistance of the host may be influenced by its genotype and the environment [31].

Few data are available in the literature regarding the photosynthetic mechanisms involved in ISR during plant growth promoting rhizobacteria (PGPR) plant–virus interactions [22,32]. The plant physiological mechanisms affected by the diseases attributed to Begomovirus include the electron transport rate (ETR), the effective photochemical quantum yield of PSII (Φ_PSII_), the maximum photochemical quantum yield of PSII (chlorophyll fluorescence: F_v_/F_m_), the potential activity of PSII (F_v_/F_0_), the CO_2_ assimilation rate (photosynthesis: A_N_), the transpiration rate (E), and the stomatal conductance (g_s_) [25,33].

Virus-infected plants reduce their photosynthetic rate due to a lower efficiency of PSII [34]. The presence of the virus capsid protein in chloroplasts causes an inhibition of PSII [35]. Tomato and cucumber plants infected with CMV and apricot plants infected with *Pox plum virus* (PPV) had decreased Φ_PSII_ [7,9]. However, our results showed that, despite virus infection, the *C. chinense* plants inoculated with *Bacillus* spp. had a healthy photosynthetic mechanism, because both the photochemical (ETR, Φ_PSII_, and F_v_/F_m_) and gas exchange (A_N_ and WUE) parameters increased.

It is known that some *Bacillus* increase photosynthesis activity to stimulate the production of endogenous sugars and regulate the plant energy acquisition, also stimulating the endogenous hormonal production (auxins, abscisic acid, jasmonic acid) [36,37]. A previous study in Habanero pepper plants inoculated with *Bacillus* spp. showed an increase in the overall photosynthetic capacity in vivo. Based on ETR, Φ_PSII_, and F_v_/F_m_ data, it was suggested that inoculation with PGPR could prepare the plant for future abiotic stress situations [25]. However, according to our studies on photochemical parameters (ETR, Φ_PSII_m and F_v_/F_m_), the inoculation with PGPR could prepare the plant for both abiotic and biotic stress situations. In this way, according to Li et al. [38], the electron transport rate is inhibited under stress conditions (i.e., there is a decrease in ATP and NADPH production). Moreover, it was suggested that a chlorophyll fluorescence (F_v_/F_m_) value of around 0.83 is optimal for most plant species [39]. Thus, the improvement of Ethe TR and F_v_/F_m_ parameters appears related to the beneficial effect of the *Bacillus* spp. on PSII.

In addition, the results showed that *C. chinense* plants infected by PepGMV and inoculated with *Bacillus* spp. isolates increased the positive effects on the photosynthetic rates with respect to infected and control plants. In this way, it was reported that the size of stem cells decreased in tobacco plants infected with CMV. However, when infected plants were inoculated with *Paenibacillus lentimorbus*, the stem cells were in a turgid state [22]. Moreover, the bacteria-inoculated plants showed few structural anomalies in the leaf tissues and increased polyphenol accumulation in the hypodermis layer, extending to the collenchyma cells. The rhizobacteria–plant interaction also yields additional CO_2_ in the roots that can be transported to the shoot [40] for use in photosynthesis [41]. The *Bacillus* spp. used in this study likely produced endogenous sugars or CO_2_ from roots as a supply of carbon for photosynthesis from the vascular system and not from the stomata. The CO_2_ assimilation rate then increased and both the stomatal conductance and transpiration rate decreased, increasing the water use efficiency. Moreover, an increase in photosynthesis is a sign of surplus in carbon assimilates, thus photosynthate surplus is probably used in other metabolic pathways, such as polyphenols production, which could decrease the severity of disease symptoms.

According to the results, the seed inoculation with *B. subtilis* K47 kept the photosynthetic apparatus of the plant healthy during the disease progression, producing larger fruits when compared with those of healthy plants. The inoculation of *Bacillus subtilis* BEB-13bs in *Lycopersicon esculentum* Mill. favored the fruit quality by increasing the size and texture [42]. *B. subtilis CAS15* reduced the incidence of *Fusarium* sp. in pepper [43], increasing the yield and fruit weight. The inoculation of *B. amyloliquefaciens* 5B6 in pepper leaves infected with CMV protected the infected plants that showed the same yield as healthy plants without rhizobacteria, suggesting an increase in ISR [44].

In this study, the inoculation with *B. subtilis* K47 decreased the viral disease severity and improved the yield, despite the PepGMV infection. During the interaction between *Bacillus subtilis* K47 and *Capsicum chinense*-PepGMV, the photosynthate surplus was likely used in the fruit production. On the other hand, it was observed that the M9 and K46 isolates had a different effect, as they did not improve the yield in plants but diminished the PepGMV severity.

Therefore, we suggest that inoculation with *Bacillus* spp. may likely promote ISR in plants infected with PepGMV, increasing the photosynthetic parameters and decreasing the viral disease severity, thus promoting a greater yield. These bacteria may thus be implemented in disease management programs in sustainable agriculture.

## 4. Materials and Methods

### 4.1. Plant Material and Growth Conditions

The H-224 population [45] of *Capsicum chinense* was used, and the seeds were disinfected with sodium hypochlorite (2%) and washed three times with sterile distilled water. Germination trays of 200 cavities filled with sterile peat moss substrate were moistened to field capacity with sterile distilled water. The seeds were then sown according to the experimental design. All trays were placed in a growth room at 25 ± 2 °C, with a photoperiod of 16/8 light/darkness, watered every second day, and leaf fertilized (UltraFol™, Biochem systems S.A. de C.V. Querétaro, Qro. México) weekly at a dose of 1 g L^−1^ distilled water. After 18 days of germination, the seedlings were transferred to Styrofoam cups (500 mL capacity filled with sterile peat moss substrate) and maintained in the same growth room under controlled conditions.

### 4.2. Bacillus spp. Isolates and Inoculation

*Bacillus cereus* K46, *Bacillus* M9 (a mixture of *B. subtilis* and *B. amyloliquefaciens*), and *B. subtilis* K47 were isolated from soil in the Yucatan Peninsula, Mexico. The growth of the isolates, the cell concentration (10^8^ cells mL^−1^ in 10 mL saline 8%), and the inoculation were performed as previously described [25].

### 4.3. Biolistic Infection

Habanero pepper (*C. chinense*) seedlings obtained from seeds inoculated with the isolates described above, and having 3–4 true leaves (30 days after germination), were infected with PepGMV by biolistics. The third and fourth leaf above the inoculation point in *C. chinense* seedlings were biolistically inoculated at a 2 cm distance from the habanero pepper seedlings and at 100 to 120 psi helium pressure with 1μm gold particles (BioRad, Hercules, CA, USA) covered with 5 μg of DNA from each of the A and B hemidimers of the PepGMV genome, as described previously [3]. The treatments were: (1) *Bacillus subtilis* (K47 isolated) + PepGMV; (2) *Bacillus cereus* (K46 isolate) + PepGMV; (3) *Bacillus* spp. (M9 isolates; a mixture of *B. subtilis* and *B. amyloliquefaciens*) + PepGMV; (4) uninoculated and noninfected plants (control); (5) plants infected with PepGMV. Each treatment consisted in one plant as experimental unit with 30 replicates. Plants with PepGMV were grown under controlled conditions (25 ± 2 °C, and photoperiod of 16/8 light/darkness) for up to 28 days post infection (dpi) with PepGMV. At 28 dpi, the plants were transferred to black polyethylene bags (400 gauge) with a 5 kg capacity (35 cm diam. and 40 cm high) filled with sterile substrate and maintained in a greenhouse under controlled conditions (30 ± 2 °C, 65 ± 3% HR and 1100 µmoles luminous intensity).

### 4.4. Virus Detection

PepGMV infection in *C. chinense* plants was evaluated using 100 ng of total DNA isolated from leaves from systemic infected individuals. The total DNA extraction was performed according to Doyle and Doyle [46] without modifications. The simples were treated with RNaseI and the DNA yield was measured spectrophotometrically (Nanodrop 2000, ThermoFisher). The PCR reactions were performed in a thermocycler (TC-412, Techne, Bibby Scientific Ltd., NJ, USA) with the following primers: 5-GCCTTGTGGAGAGCTAATGC-3 and 5- TTAGCGCAGTTGATGTGGAG-3 (213 bp) to target the *AC2* gene. The PCR conditions were: 1 cycle at 94 °C (5 min); 35 cycles at 94 °C (30 s), 58 °C (30 s), and 72 °C (30 s); and a final extension at 72 °C (10 min).

### 4.5. Symptoms Severity

The severity caused by PepGMV was evaluated at 9 and 15 dpi in a growth room under controlled conditions, and at 200 dpi in a greenhouse. A four-level severity scale was modified and a methodology was used with values: 1: Golden mosaic; 2: Golden mosaic and leaf distortion; 3: Golden mosaic, leaf distortion, and chlorosis; 4: Golden mosaic, leaf distortion, chlorosis, and leaf curl; 5: Golden mosaic, general yellowing leaf distortion, chlorosis, and leaf curl. The severity of symptoms was calculated with a mean of ten observations per treatment [47].

### 4.6. Chlorophyll Fluorescence and Gas Exchange Analysis

The photochemical parameters of the leaves were measured with a portable pulse amplitude modulation fluorometer (PAM; Walz, Effeltrich, Germany) at pre-dawn (4:30 h) in plants 140 days after sowing. The light response curves of both the relative electron transport rate (ETR_PSII_) and the effective photochemical quantum yield (*Φ*_PSII_) of PSII were obtained with actinic irradiance (PPFD) from 0 to 1500 µmol photons m^−2^ s^−1^. Both the maximum photochemical quantum yield of photosystem II (F_v_/F_m_) and the potential activity of PSII (F_v_/F_0_) were measured: F_v_ = variable fluorescence (F_m_–F_0_), F_0_ = initial fluorescence, and F_m_ maximum fluorescence. A saturating pulse once every 20 s was applied to obtain the steady-state fluorescence, as well was photochemical fluorescence quenching (qP). All the calculated parameters and light characteristics used in the fluorometer (saturation pulse intensity, pulse length, and light intensity) were as described [25]. Gas exchange analyses were carried out under greenhouse conditions (average temperature: 28 °C during the day and 24 °C during the night; photosynthetic active radiation: 1200 µmol photons m^−2^ s^−1^ at noon; average HR: 65%) using a portable infrared gas analyzer system (IRGA; LICOR, LI–6400, Nebraska, USA). Fifteen fully expanded young leaves from each treatment were placed in the IRGA gas-exchange leaf chamber. The CO_2_ assimilation rate (*A_N_*), stomatal conductance (*g_s_*), transpiration (*E*), and water use efficiency (WUE) were measured as described [48].

### 4.7. Agronomic Parameters

For the evaluation of the yield and fruit characteristics, the harvest was started from week 12 after transplanting to the greenhouse (datg). A harvest was carried out every seven days (week 12, 13, and 14 datg), and the completely ripe fruit (orange) per plant were weighed and quantified per harvest (three harvests in total).

### 4.8. Experimental Design and Statistical Analysis

The experiments were performed in a completely randomized design. The photosynthetic, gas exchange, and agronomic parameters were examined through ANOVA and the means of each treatment were compared with Tukey’s HSD test at *p* ≤ 0.05 (Statistic Six Sigma, Release 7, StatSoft).

## Figures and Tables

**Table 1:** Infectivity, viral detection, and severity in pepper plants inoculated with *Bacillus.* spp. isolates K47, K46, M9, and PepGMV-infected plants; plants with PepGMV only; and control plants (bacteria not inoculated and PepGMV not infected). Different letters represent statistically significant differences (Tukey, α = 0.05, *n* = 5).

Treatments	PCR Detection	Infected/Bombarded	Severity Scale		
			9 dpi	15 dpi	200 dpi
K47 + PepGMV	+	5/5	0.2 ^b^	1.2 ^b^	1.3 ^b^
K46 + PepGMV	+	5/5	0.6 ^a,b^	1.1 ^b^	4.5 ^a^
M9 + PepGMV	+	5/5	0.2 ^b^	1.1 ^b^	3.1 ^a^
Control	-	0/0	0.0 ^b^	0.0 ^c^	0.0 ^b^
PepGMV	+	5/5	1 ^a^	2.5 ^a^	4.8 ^a^

**Table 2:** Production of Habanero pepper (*C. chinense*) plants inoculated with *Bacillus subtilis* K47, *B. cereus* spp. K46, *Bacillus* sp. M9; plants infected with PepGMV; and PepGMV-infected plants. Control = not inoculated with bacteria and not infected with PepGMV. Different letters represent statistically significant differences (Tukey, α = 0.05, *n* = 30).

Treatment	Fruits per Plant	Mean Fruit Weight (g)	Yield (g) per Plant
K47 + PepGMV	56 ± 1.1 ^a^	8.0 ± 0.03 ^a^	452 ± 7.9 ^a^
K46 + PepGMV	46 ± 1.0 ^c^	7.8 ± 0.04 ^b^	359 ± 6.8 ^b^
M9 + PepGMV	40 ± 1.0 ^d^	7.8 ± 0.04 ^b^	311 ± 7.8 ^c,d^
Control	52 ± 1.4 ^b^	6.2± 0.03 ^c^	321 ± 7.4 ^c^
PepGMV	52 ± 1.0 ^b^	5.6± 0.04 ^d^	295 ± 5.4 ^d^

## Data Availability

Not applicable.
